# A realistic human model to teach physicians how to diagnose ascites using point-of-care ultrasound

**DOI:** 10.1590/2175-8239-JBN-2023-0034en

**Published:** 2023-07-24

**Authors:** Marcus G. Bastos

**Affiliations:** 1 Universidade Federal de Juiz de Fora, Programa de Pós-Graduação em Saúde, Juiz de Fora, MG, Brazil.; 2 Faculdade de Ciências Médicas e da Saúde, Juiz de Fora, MG, Brazil.; 3 Centro Universitário Governador Ozanam Coelho, Faculdade de Medicina, Ubá, MG, Brazil.

**Figure 1.**
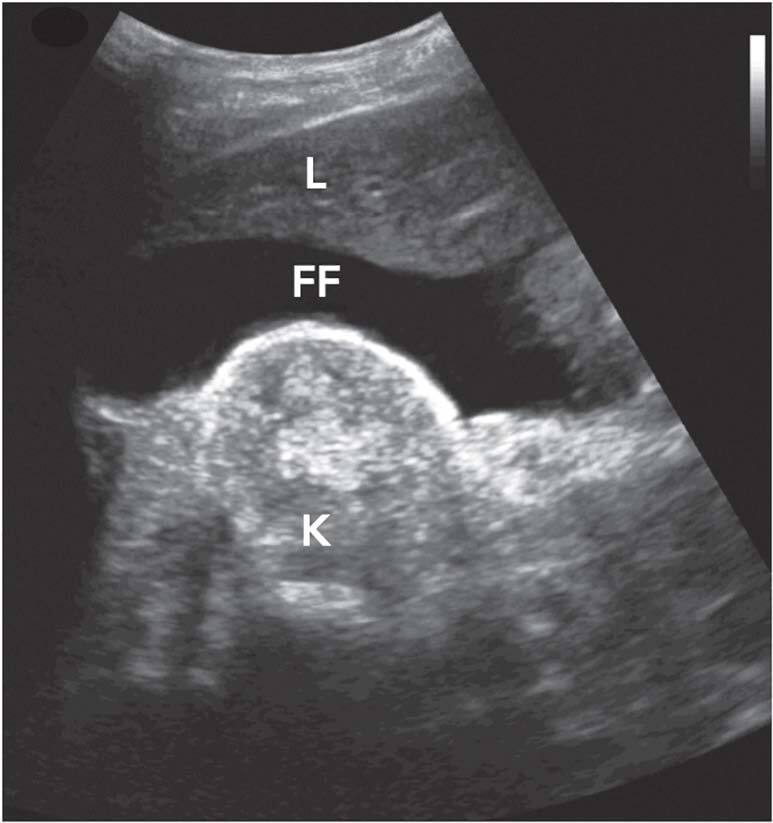
Anechoic image in the right upper quadrant showing free fluid in Morrison’s pouch, acquired from a patient with two liters of dialysate in the peritoneal cavity. L: liver; K: kidney with signs of chronic disease; FF: free fluid.

Dear Editor,

The use of point-of-care ultrasound (POCUS) relies on three words beginning with the letter I: 1. Insonation, or the acquisition ultrasound images; 2. Interpretation, when the attending physician interprets the acquired images during a patient encounter; and 3. Integration, when the new clinical data is added to the knowledge previously acquired via investigation, palpation, percussion, and auscultation, thus enhancing the accuracy of physical examination. Ascites is a relatively common finding in patients with kidney disease in a variety of clinical contexts^1^ and its diagnosis should be included in POCUS training programs in nephrology^2^. Actors are often used in POCUS training programs to simulate different clinical situations. In this human model of ascites simulation, we have used clinically stable patients on peritoneal dialysis (PD) in our training sessions. On the day of the hands-on session, the patient is advised not to drain the peritoneal cavity after the last PD cycle, thus keeping approximately 2 liters of intraperitoneal dialysate. We use a convex low-frequency ultrasound transducer (2–5 MHz) connected to portable ultrasound equipment (VERSANA ACTIVE, GE) and insonation is performed with the actor in dorsal decubitus. The windows used in the identification of the anechoic image corresponding to free intraperitoneal fluid are the right upper quadrant (Figure 1), pelvis, and left upper quadrant. This protocol allows the representation of ascites in the described windows in 100% of the simulations performed. In summary, the use of patients undergoing dialysis with dialysate in the abdominal cavity provides a realistic model for simulating ascites in point-of-care ultrasound training in undergraduate and graduate medical education programs.
